# Intramedullary hemorrhage caused by spinal cord hemangioblastoma: a case report

**DOI:** 10.1186/1756-0500-7-823

**Published:** 2014-11-20

**Authors:** Masao Koda, Chikato Mannoji, Takashi Itabashi, Tsuneji Kita, Masazumi Murakami, Masashi Yamazaki, Masaaki Aramomi, Osamu Ikeda, Takeo Furuya

**Affiliations:** Department of Orthopedic Surgery, Chiba Aoba Municipal Hospital, 1-8-1, Inohana, Chuo-K, Chiba, 260-8670 Japan; Department of Orthopedic Surgery, Chiba University Graduate School of Medicine, Chiba Aoba Municipal Hospital, Chiba, Japan; Department of Orthopedic Surgery, Narita Red Cross Hospital, Chiba, Japan; Department of Orthopedic Surgery, University of Tsukuba, Tsukuba, Japan

**Keywords:** Hemangioblastoma, Intramedullary hemorrhage, Spinal cord

## Abstract

**Background:**

Hemorrhage caused by spinal cord hemangioblastoma is rare, usually presenting as a subarachnoid hemorrhage. Intramedullary hemorrhage is an extremely rare manifestation of spinal cord hemangioblastoma.

**Case presentation:**

Forty-year-old Japanese male patient presented with acute paraplegia. Magnetic resonance (MR) imaging of the spinal cord revealed intramedullary hemorrhage. An intramedullary mass lesion was detected at the 8th thoracic vertebral level (T8) in a gadolinium enhanced-MR image. Spinal angiography revealed an intramedullary tumor stain at the level of T8. Therefore we diagnosed the problem as intramedullary hemorrhage caused by the hemangioblastoma. One month after the onset, extirpation of the intramedullary hemangioblastoma was performed. The tumor was completely removed. Pathological findings revealed a typical hemangioblastoma. At his final follow-up visit, the patient showed no apparent neurological recovery.

**Conclusion:**

Hemangioblastoma can be a cause of intramedullary hemorrhage should be considered in such cases.

## Background

Hemorrhage caused by spinal cord hemangioblastoma is rare, usually presenting as a subarachnoid hemorrhage [1]. Intramedullary hemorrhage is an extremely rare manifestation of spinal cord hemangioblastoma [2, 3]. The present report describes a patient who presented with an acute intramedullary hemorrhage caused by a hemangioblastoma in the thoracic spinal cord, resulting in immediate paraplegia. Although subsequent tumor extirpation was performed, the patient showed only a slight neurological recovery.

## Case presentation

A 40-year-old Japanese male patient presented with acute paraplegia after extending his trunk during bathing. Before onset, he was doing well without any paralysis. He had untreated diabetes. The patient was admitted to a local hospital and then transferred to our department for surgery.

**Figure 1:**
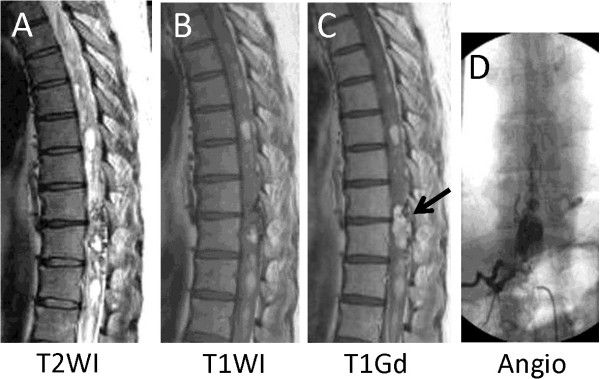
**Preoperative imaging.** Magnetic resonance (MR) imaging of the spinal cord revealed a syrinx extending from the bulbs to the lumbar spinal cord, and intramedullary hemorrhage **(A, B)**. An intramedullary mass lesion was detected at the level of 8th thoracic vertebra (T8) in a gadolinium enhanced-MR image (**C**, arrow). Spinal angiography revealed an intramedullary tumor stain at T8 with a feeding artery branch from an intercostal artery and a dilated and tortuous draining vein **(D)**.

On admission, the patient was awake and alert, and did not complain of either headache or neck pain. There were no signs or family history of von Hippel–Lindau disease. Initial physical examination revealed that muscle strength and sensation of his upper extremities were normal. There was complete loss of sensation to pinprick below the 7th thoracic vertebral level (T7) including the perineal region; however, touch sense was preserved except for the feet bilaterally. There was complete motor loss in his right lower extremity and severe weakness in his left lower extremity, which had a manual muscle testing grade of from 1 to 2. Patellar and Achilles tendon reflexes were absent bilaterally. There was no apparent Babinski reflex.Magnetic resonance (MR) imaging of the spinal cord revealed a syrinx extending from the bulbs to the lumbar spinal cord, and intramedullary hemorrhage (Figure 1A,B). An intramedullary mass lesion was detected at the level of T8 in a gadolinium enhanced-MR image (Figure 1C, arrow). Spinal angiography revealed an intramedullary tumor stain at the level of T8 with a feeding artery branch from an intercostal artery into the tumor and a dilated and tortuous draining vein mainly located on the dorsal surface of the spinal cord (Figure 1D).

Therefore we diagnosed the problem as intramedullary hemorrhage caused by the hemangioblastoma. We planned to perform tumor extirpation as elected surgery in the subacute stage of hemorrhage, and we decided to wait for at least 3 to 4 weeks after the onset.

After admission, the patient showed slight motor and sensory recovery. The patient was treated conservatively with physiotherapy under hospitalization. One month after the onset, he could extend his right knee and ankle, and he recovered sensation to pinprick on his trunk.

One month after the onset, the patient underwent surgery. Under general anesthesia, a T7–T9 laminectomy followed by extirpation of the intramedullary hemangioblastoma was performed. The tumor was completely removed. Pathological findings revealed a typical hemangioblastoma. At his final follow-up visit, the patient showed no apparent neurological recovery.

## Discussion

Here we report a case of acute intramedullary hemorrhage caused by hemangioblastoma of the thoracic spinal cord. Despite complete removal of the tumor, the patient obtained only slight motor and sensory recovery.

Previous reports describing intramedullary hemorrhage caused by spinal cord hemangioblastoma showed acute paraplegia after the onset, and poor recovery even after complete removal of the tumor. Preexisting intramedullary hemangioblastoma might cause subclinical spinal cord damage resulting in additive catastrophic damage caused by intramedullary hemorrhage.

Whether a better outcome can be obtained by emergent tumor removal remains unclear. Steiger *et al.* reported the clinical outcome of conservative and surgical treatment for intramedullary cavernous hemangioma with hemorrhage, and indicated that the intramedullary tumor removal operation presents a risk of additional damage to the acutely paralyzed spinal cord [4]. In this context, we waited for approximately 4 weeks after the onset to perform surgery. However, it is still controversial whether the emergent tumor removal or elective surgery results in better outcome for intramedullary hemorrhage caused by hemangioblastoma.

Once bleeding occurs, it leads to a catastrophic sequela of paralysis that not even tumor extirpation can rescue. Tumor removal surgery should be considered for asymptomatic hemangioblastoma or hemangioblastoma with only slight symptoms. At least, the patient should be informed of the possibility that hemangioblastoma can cause intramedullary hemorrhage.

## Conclusion

In conclusion, that hemangioblastoma can be a cause of intramedullary hemorrhage should be considered in such cases.

## Consent

Written informed consent was obtained from the patient for publication of this Case Report and accompanying images. A copy of the written consent is available for review by the Editor-in-Chief of this journal.

## Abbreviations

T: Thoracic spine
MR: Magnetic resonance.
